# The Bounce-back Effect: What Happens After Cessation of Low-dose Semaglutide in People With HIV

**DOI:** 10.1093/cid/ciaf652

**Published:** 2025-11-26

**Authors:** Kristine M Erlandson, Douglas W Kitch, Amy Kantor, Pablo F Belaunzaran-Zamudio, Todd T Brown, Carl J Fichtenbaum, Sonya L Heath, Fred R Sattler, Jordan E Lake

**Affiliations:** Department of Medicine, University of Colorado Anschutz Medical Campus, Aurora, Colorado, USA; Harvard TH Chan School of Public Health, Boston, Massachusetts, USA; Harvard TH Chan School of Public Health, Boston, Massachusetts, USA; Contractor for National Institute of Allergy and Infectious Diseases, Rockville, Maryland, USA; Department of Medicine, Johns Hopkins University School of Medicine, Baltimore, Maryland, USA; Department of Medicine, University of Cincinnati, Cincinnati, Ohio, USA; Department of Medicine, University of Alabama Birmingham, Birmingham, Alabama, USA; Deparment of Medicine, University of Southern California Keck School of Medicine, Los Angeles, California, USA; Department of Internal Medicine, UTHealth, Houston, Texas, USA

**Keywords:** GLP-1 receptor agonist, weight regain, MASLD, HIV

## Abstract

**Background:**

We previously reported reductions in weight and cardiometabolic risk factors in people with human immunodeficiency virus (PWH) receiving semaglutide; here, we explored the durability of these changes after treatment cessation.

**Methods:**

ACTG A5371 enrolled PWH ≥18 years on suppressive antiretroviral therapy with metabolic dysfunction–associated steatotic liver disease. All received subcutaneous semaglutide 1 mg weekly for 24 weeks followed by 24 weeks off semaglutide. We measured weight and cardiometabolic risk factors (blood pressure, cholesterol, metabolic syndrome) at weeks 0, 24, and 48. Mean (95% confidence interval [CI]) changes were estimated using linear regression.

**Results:**

The 49 participants had a median age of 52 years, body mass index 35 kg/m^2^, 39% Hispanic and 33% Black, and 43% female. After the mean 7.8 kg (95% CI, 6.1–9.5) weight loss in the first 24 weeks, absolute mean weight regain from 24 to 48 weeks was +2.9 kg (95% CI, 1.5-4.3). Weight regain was accompanied by significant increases in waist circumference (2.0 cm [0.9-3.1]) and fasting glucose (5.1 mg/dL [0.9-9.3]) without significant changes in blood pressure, total or low-density lipoprotein cholesterol, triglycerides, or metabolic syndrome.

**Conclusions:**

Short-term, low-dose, semaglutide was associated with cardiometabolic benefit in PWH, but rapid weight regain and some loss of cardiometabolic benefit occurred after stopping semaglutide, similar to the general population. Further study of higher dose semaglutide and strategies to maintain benefits following initial therapy are needed in PWH.

**ClinicalTrials.gov:** NCT04216589

## Teaser

Short-term, low-dose, semaglutide was associated with initial cardiometabolic benefit, but rapid weight regain and some loss of cardiometabolic benefit occurred after stopping semaglutide. Further study of strategies to maintain benefits following initial therapy are needed.

## Graphical Abstract

**Graphical Abstract:**
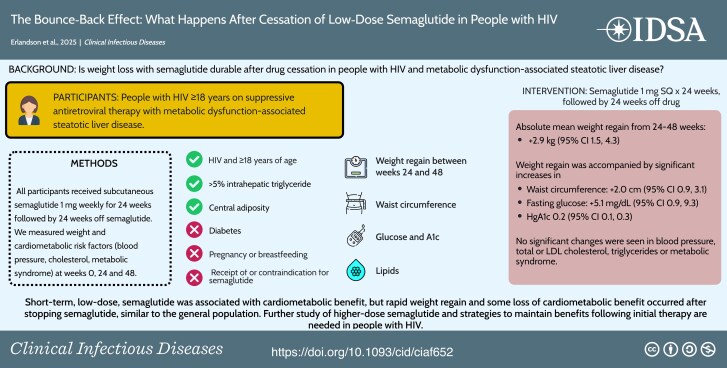
This graphical abstract is also available at Tidbit: https://tidbitapp.io/institutional-portal/clinical-infectious-diseases/tidbits/the-bounce-back-effect-what-happens-after-cessation-of-low-dose-semaglutide-in-people-with-hiv/update

## BACKGROUND

Newer weight loss agents, including glucagon-like peptide-1 receptor agonists (GLP-1 RA [eg, semaglutide]), and combined GLP-1 RA/glucose-dependent insulinotropic polypeptide (eg, tirzepatide), have revolutionized treatment of obesity [1]. Emerging data on additional benefits of these agents (heart failure, arthritis, Parkinson disease, addiction), suggest a broad role for obesity and nonobesity-associated conditions [2]. The additional benefits appear to result from mechanisms beyond the weight-reducing capabilities of these agents, such as reduced inflammation, and may be related to the presence of GLP-1 receptors in multiple human tissues, including in the brain.

However, initial studies suggest that the beneficial effects of these agents remain only while taking the therapy. For example, in the STEP 1 study of adults with obesity who lost a mean 17% of weight with 68 weeks of semaglutide 2.4 mg weekly, participants regained two thirds of prior weight lost and most cardiometabolic improvements (blood pressure, hemoglobin A1c [HbA1c]) returned to baseline values during 52 weeks after drug cessation [3]. In the STEP 4 study, participants were randomized to continue semaglutide versus placebo after completing 20 weeks of induction with semaglutide 2.4 mg weekly, with a mean weight loss of 10.6%. The placebo group regained 6.9% of body weight after an additional 48 weeks of follow-up [4]. A recent meta-analysis including the STEP 1 and STEP 4 trials and a tirzepatide trial reported a partial weight regain of 9.7 kg following cessation of therapy (after 48–52 weeks of initial therapy), regardless of lifestyle interventions [5].

Little is known about weight regain effects in people with human immunodeficiency virus (PWH), who have increasing rates of obesity, but may experience differences in body composition compared to the general population [6]. Obesity-related conditions such as metabolic dysfunction–associated steatotic liver disease (MASLD) occur more commonly in PWH and may occur at a lower body mass index (BMI) [7]. To specifically investigate the role of GLP-1 RA on MASLD, in the SLIM LIVER Study, we tested lower dose semaglutide (1 mg weekly) for 24 weeks in PWH and MASLD [8]. We observed a 35% reduction in our primary outcome of intrahepatic triglyceride (IHTG) content (magnetic resonance imaging–measured liver fat) over 24 weeks; we also found reductions in weight (mean −7.8 kg; 95% confidence interval [CI], 6.1–9.5), fasting glucose, and serum triglycerides. As this study tested a lower dose of semaglutide in a unique population (PWH) with MASLD, we hypothesized that some improvements in the metabolic components underlying liver steatosis might persist following treatment cessation. In this subsequent pilot study, we assessed changes in weight, waist circumference, lipids, and glucose 24 weeks after discontinuation of semaglutide.

## METHODS

### Study Design

ACTG A5371 SLIM LIVER was a phase IIb, single-arm, open-label pilot study on the effects of low-dose semaglutide on IHTG in PWH and MASLD conducted at 9 U.S. sites (NCT04216589) [8]. Each study site obtained local institutional review board approval. All participants provided written informed consent before enrollment in the study. The primary endpoint was 24-week change in IHTG; prespecified analyses reported here include changes at 48 weeks, following 24 weeks of treatment cessation.

### Participants

PWH were enrolled from February 2021 to September 2022. Inclusion criteria included being age 18 years and older, ≥ 5% IHTG content by magnetic resonance imaging proton-derived fat fraction, and central adiposity (defined by a minimum waist circumference [WC] of ≥95 cm for individuals assigned male sex at birth or ≥94 cm for individuals assigned female sex at birth). Participants also had to meet at least 1 of the criteria for insulin resistance or prediabetes: fasting plasma glucose 100–125 mg/dL, HbA1c between 5.7% and 6.4%, or homeostatic model assessment for insulin resistance > 3.0 [9].

### Intervention

Participants self-administered semaglutide subcutaneously into the abdomen, thigh, or upper arm, starting at 0.25 mg/week and titrating up to 1.0 mg/week at week 4. Treatment continued for a total of 24 weeks. Participants were followed for 24 weeks after semaglutide cessation and instructed to remain on their same antiretroviral therapy regimen over the course of the study.

### Study Evaluations and Outcomes

Demographics, medical history, and current and recent medication history were provided by participant self-report and verified by medical record review, when available. Physical activity was self-reported by the International Physical Activity Questionnaire (short form), and quality of dietary intake was collected by a 9-question nutrition survey, based on the Rapid Eating Assessment for Participants. CD4^+^ T-lymphocyte counts were performed at local certified laboratories. HIV-1 RNA was measured at protocol-designated laboratories in the United States using the Abbott real-time HIV-1 assay (lower limit of detection 40 copies/mL).

The following study outcomes were measured at weeks 0, 24, and 48: Weight and WC measurements were performed according to standardized ACTG protocols. BMI was calculated in kg/m^2^. Fasting (≥8 hours) metabolic panel included glucose and liver enzymes, HbA1c, lipid profiles with low-density lipoprotein, triglyceride, and high-density lipoprotein cholesterol measurement.

### Statistical Analysis

The analysis (per protocol) population included all participants who remained on study treatment until at least 28 days before the week 24 imaging, did not have eligibility violations, and did not start prohibited medications known to cause weight change prior to week 24. Continuous participant characteristics were summarized using median and interquartile range. Categorical characteristics were summarized using frequency and percentage. Change from baseline to weeks 24 and 48 and change from weeks 24 to 48 were estimated with a linear regression model without an intercept term to estimate means and 95% CI. Subgroup analyses were performed for sex at birth, gender, race/ethnicity, age, and weight loss responder status (weight loss of at least 2.27 kg or 5 pounds between weeks 0 and 24, an amount beyond that expected from day-to-day variability and changes in water weight alone [10]). Among weight loss responders, participants with and without significant weight regain (≥2.27 kg or 5 pounds) from weeks 24 to 48 were compared with Wilcoxon rank sum and Fisher's exact tests. Statistical analysis was performed by SAS software (Version 9.4 for Linux. Copyright 2016 by SAS Institute Inc., Cary, NC, USA). No adjustments for multiple comparisons were made.

## RESULTS

### Baseline Characteristics

Among the 51 participants initially enrolled, 49 remained on study at week 24, and 47 remained on study at week 48. At baseline, as previously published [8], the median age was 52 years, BMI 35 kg/m^2^, 39% were of Hispanic ethnicity, 33% of Black/African American race, and 43% were cis/trans female. The majority (82%) were using INSTI-based antiretroviral therapy and all had HIV-1 RNA <40 copies/mL.

#### Body Composition and Cardiometabolic Risk Factors Changes After Semaglutide Cessation

**Figure 1.**
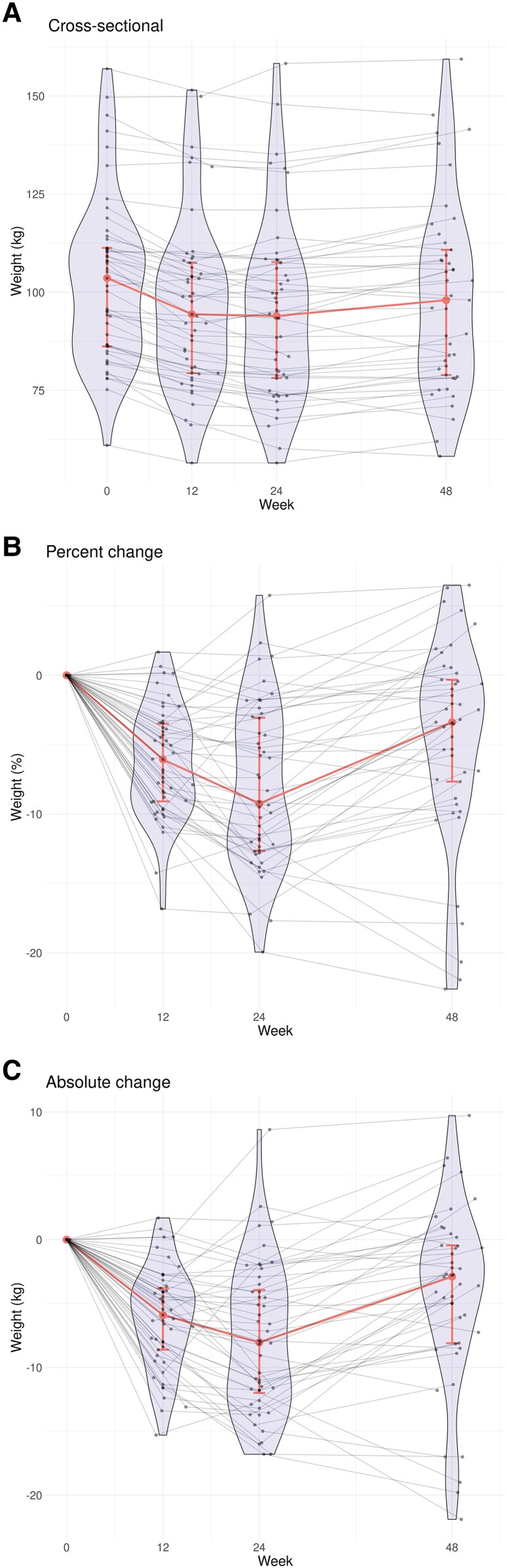
Weight changes during and following semaglutide treatment by *A*, cross-sectional measurements at each time point; *B*, percent change between baseline, week 12, week 24, and week 48; and *C*, absolute change between baseline, week 12, week 24, and week 48. At each study week, individual participant measurements are shown as points and are connected with gray lines to summarize within-participant changes. Violin plots illustrate the distribution and density of the cross-sectional data. Medians and interquartile ranges are shown in red, with the medians joined to depict overall trend.

For our primary endpoint for this analysis, weight regain between weeks 24 and 48, we found the absolute mean weight regain among all participants was +2.9 kg (95% CI, 1.5–4.3); distribution and variability of observed data are shown in Figure 1. Overall, although more than half (52% [23/44]) of participants regained at least 2.27 kg, 60% (27/45) of participants maintained an overall mean weight loss of at least 2.27 kg by week 48 compared to baseline.

Cessation of treatment was also associated with significant increases in mean WC (2.0 cm [95% CI, .9–3.1]), fasting glucose (5.1 mg/dL [95% CI, .9–9.3]), and HbA1c (0.2 [95% CI, .1–.3]).

Differences in change in weight, WC, glucose, and HbA1C were explored by sex, race/ethnicity, age, and by initial weight loss. No between-group differences reached statistical significance, but the study was not powered for these subgroup analyses (Supplementary Tables 1–4).

We found no significant changes in fasting serum total cholesterol, low-density lipoprotein cholesterol, and high-density lipoprotein cholesterol, triglycerides, systolic blood pressure, or metabolic syndrome though trends were generally toward reversal of the initial beneficial effects (Supplementary Figure 1).

#### Factors Potentially Affecting Weight Regain vs Maintenance

Overall, we observed improvements in the derived diet summary score (0 = best diet quality to 27 = worst) at week 24 (−1.42 [−2.45 to −0.38]; *P* = .008) that were maintained at week 48 (−1.14 [−2.20 to −0.08]. We observed minimal changes in self-reported physical activity between weeks 0 and 48, including in walking time (94 [95% CI, −380 to 567] metabolic equivalent minutes/week, *P* = .7), moderate-intensity activity (−202 [−766 to 361] metabolic equivalent minutes/week, *P* = .47), or vigorous intensity activity (89 [−416 to 594], *P* = .72).

We next focused on the 34 participants who had a > 2.27-kg decrease in the initial 24 weeks and then either had a significant regain or maintained: 20 (59%) regained >2.27 kg and 14 (41%) did not (Supplementary Table 5). Among those with initial weight loss, participants with greater weight regain (>2.27 kg weight regain (vs ≤2.27 kg regain) had significantly greater self-reported physical activity (moderate and total) at baseline and at week 24, with no significant difference in activity changes between week 0 to 24 or 24 to 48. Dietary patterns and other demographic characteristics were not significantly different by weight regain groups.

## DISCUSSION

Among people with HIV and MASLD, as hypothesized, we found that cessation of low-dose semaglutide (1 mg weekly) was associated with rapid weight regain of over 5 pounds (>2.27 kg), and accompanied by increases in WC, glucose, and HbA1c (despite the population not having diabetes). Although changes in other cardiometabolic measures were not significantly different between week 24 and week 48, these measures tended toward a reversal of the initial beneficial effects. These results are generally consistent with the reversal of weight loss effects seen in populations without HIV and highlight the chronic nature of obesity and need for long-term interventions.

Variability in weight, even within the setting of clinical trials, is highlighted by the individual weight trajectories in Figure 1. Although the majority of participants with initial weight loss experienced weight regain after cessation, nearly one quarter continued to have at least small declines in weight after cessation. Although our sample size was too small for meaningful comparisons, the male predominance in this group of continued weight loss is notable, with lower weight regain among males similarly reported in the STEP 1 Trial [3]. Incorporating lifestyle change is essential in ongoing weight maintenance, including sustainable physical activity and healthy nutrition [11, 12], and have been shown to limit weight regain either alone or in combination with GLP-1 RA therapy [13]. Notably, those with greater weight regain were more active at baseline, week 24, and week 48 compared to those with a ≤2.27-kg regain. This group may have had a more profound metabolic slowing in the setting of weight loss [14] and struggled to maintain weight even with physical activity. These findings underscore the complexity in obesity management and the need for an individualized approach to weight management.

Novel obesity medications, especially semaglutide and tirzepatide, are highly effective in mitigating the complications of obesity, including cardiovascular disease [15–21]. Despite the success of these therapies, adherence for more than 1 year remains low [22]. Prospective, randomized studies investigating the efficacy and acceptability of off-ramping, micro-dosing, or intermittent-dosing strategies for long-term use are critically needed. Other strategies have been proposed and tested, including combination liraglutide with intensive exercise [13], switch to other medications including metformin [23], or a slow taper off of therapy [24], with some success.

Notably, in the present study the mean weight regain was less than the initial amount lost, suggesting possible benefit in a low, intermittent dosing, or extended dose intervals as a longer term strategy [25, 26].

Several limitations of our study should be acknowledged, including the small sample size, and lack of a control arm. Physical activity and dietary intake were self-reported. Our dose was lower than other studies and the duration shorter, with weight loss less than in some studies, and thus we may have observed less weight rebound. Our population was primarily U.S. based and findings may not be generalizable to the growing obesity epidemic among PWH in sub-Saharan Africa. However, the participant diversity in sex/gender, race, and ethnicity, and inclusion of a population of PWH adds to the novelty and overall generalizability of our findings.

In summary, cessation of semaglutide was associated with an expected regain in weight and loss of cardiometabolic benefit, though not all participants experienced weight regain. Further studies are needed to explore therapeutic options for maintenance of GLP-1 RA effects with lower, intermittent, or alternative dosing, combined with robust lifestyle interventions to improve long-term adherence for a chronic condition.

## Supplementary Material

ciaf652_Supplementary_Data

## Supplementary Data

Supplementary materials are available at *Clinical Infectious Diseases* online. Consisting of data provided by the authors to benefit the reader, the posted materials are not copyedited and are the sole responsibility of the authors, so questions or comments should be addressed to the corresponding author.
