# Supplementary material for: The Bounce-back Effect: What Happens After Cessation of Low-dose Semaglutide in People With HIV
Source: Clin Infect Dis. 2025 Nov 26;82(3):e544–8. doi: 10.1093/cid/ciaf652 (PMC13017447; doi:10.1093/cid/ciaf652)

Supplemental Table 1. Change in Weight by Subgroup

| **Covariate** | **Outcome** | **Subgroup** | **n** | **Estimated Effect** | **95% CI** | **P-value** |
| --- | --- | --- | --- | --- | --- | --- |
| Sex at birth | Weight (kg) Change, Week 24 to Week 48 | Female | 16 | 3.68 | (1.32, 6.04) | 0.41 |
|  |  | Male | 28 | 2.47 | (0.68, 4.25) |  |
|  | | | | | | |
| Gender | Weight (kg) Change, Week 24 to Week 48 | Cis or Trans Female | 19 | 3.42 | (1.25, 5.59) | 0.53 |
|  |  | Cis or Trans Male | 25 | 2.51 | (0.62, 4.41) |  |
|  | | | | | | |
| Race/Ethnicity | Weight (kg) Change, Week 24 to Week 48 | Black (regardless of ethnicity) | 16 | 2.61 | (0.20, 5.01) | 0.91 |
|  |  | White Hispanic | 14 | 2.81 | (0.24, 5.38) |  |
|  |  | White non-Hispanic and Other | 14 | 3.34 | (0.77, 5.91) |  |
|  | | | | | | |
| Age (years) | Weight (kg) Change, Week 24 to Week 48 | <40 | 8 | 4.56 | (1.20, 7.92) | 0.55 |
|  |  | 40-60 | 31 | 2.55 | (0.84, 4.25) |  |
|  |  | >60 | 5 | 2.49 | (-1.76, 6.74) |  |
|  | | | | | | |
| Semaglutide Adherence | Weight (kg) Change, Week 24 to Week 48 | 100% | 38 | 2.71 | (1.17, 4.24) | 0.48 |
|  |  | <100% | 6 | 4.17 | (0.30, 8.03) |  |
|  | | | | | | |
| Week 24 Weight Response | Weight (kg) Change, Week 24 to Week 48 | >2.27 kg lost | 34 | 3.38 | (1.77, 4.98) | 0.22 |
|  |  | >2.27 kg not lost | 10 | 1.31 | (-1.65, 4.26) |  |
|  | | | | | | |
| Week 24 Quartile | Weight (kg) Change, Week 24 to Week 48 | Q1 | 12 | 3.65 | (0.87, 6.43) | 0.79 |
|  |  | Q2 | 10 | 2.05 | (-1.00, 5.10) |  |
|  |  | Q3 | 10 | 2.16 | (-0.89, 5.21) |  |
|  |  | Q4 | 12 | 3.50 | (0.71, 6.28) |  |
|  | | | | | | |

Supplemental Table 2. Change in Waist Circumference by Subgroup

| **Covariate** | **Outcome** | **Subgroup** | **n** | **Estimated Effect** | **95% CI** | **P-value** |
| --- | --- | --- | --- | --- | --- | --- |
| Sex at birth | Waist Circumference (cm) Change, Week 24 to Week 48 | Female | 15 | 2.09 | (0.21, 3.98) | 0.92 |
|  |  | Male | 26 | 1.97 | (0.54, 3.41) |  |
|  | | | | | | |
| Gender | Waist Circumference (cm) Change, Week 24 to Week 48 | Cis or Trans Female | 18 | 1.88 | (0.16, 3.60) | 0.83 |
|  |  | Cis or Trans Male | 23 | 2.13 | (0.60, 3.65) |  |
|  | | | | | | |
| Race/Ethnicity | Waist Circumference (cm) Change, Week 24 to Week 48 | Black (regardless of ethnicity) | 14 | 1.42 | (-0.54, 3.38) | 0.70 |
|  |  | White Hispanic | 14 | 2.57 | (0.61, 4.53) |  |
|  |  | White non-Hispanic and Other | 13 | 2.06 | (0.03, 4.10) |  |
|  | | | | | | |
| Age (years) | Waist Circumference (cm) Change, Week 24 to Week 48 | <40 | 7 | 2.30 | (-0.43, 5.03) | 0.41 |
|  |  | 40-60 | 31 | 1.70 | (0.40, 3.00) |  |
|  |  | >60 | 3 | 4.60 | (0.42, 8.78) |  |
|  | | | | | | |
| Semaglutide Adherence | Waist Circumference (cm) Change, Week 24 to Week 48 | 100% | 35 | 2.34 | (1.13, 3.54) | 0.17 |
|  |  | <100% | 6 | 0.15 | (-2.76, 3.06) |  |
|  | | | | | | |
| Week 24 Weight Response | Waist Circumference (cm) Change, Week 24 to Week 48 | >2.27 kg lost | 31 | 2.24 | (0.93, 3.54) | 0.49 |
|  |  | >2.27 kg not lost | 10 | 1.33 | (-0.97, 3.63) |  |
|  | | | | | | |
| Week 24 Quartile | Waist Circumference (cm) Change, Week 24 to Week 48 | Q1 | 12 | 2.85 | (0.76, 4.94) | 0.41 |
|  |  | Q2 | 9 | 0.38 | (-2.03, 2.79) |  |
|  |  | Q3 | 10 | 2.71 | (0.42, 5.00) |  |
|  |  | Q4 | 10 | 1.80 | (-0.49, 4.09) |  |
|  | | | | | | |

Supplemental Table 3. Change in Fasting Glucose by Subgroup

| **Covariate** | **Outcome** | **Subgroup** | **n** | **Estimated Effect** | **95% CI** | **P-value** |
| --- | --- | --- | --- | --- | --- | --- |
| Sex at birth | Fasting Glucose (mg/dL) Change, Week 24 to Week 48 | Female | 15 | 6.40 | (-0.74, 13.54) | 0.65 |
|  |  | Male | 27 | 4.37 | (-0.95, 9.69) |  |
|  | | | | | | |
| Gender | Fasting Glucose (mg/dL) Change, Week 24 to Week 48 | Cis or Trans Female | 18 | 9.11 | (2.80, 15.42) | 0.097 |
|  |  | Cis or Trans Male | 24 | 2.08 | (-3.38, 7.55) |  |
|  | | | | | | |
| Race/Ethnicity | Fasting Glucose (mg/dL) Change, Week 24 to Week 48 | Black (regardless of ethnicity) | 14 | 1.71 | (-5.62, 9.05) | 0.39 |
|  |  | White Hispanic | 14 | 8.79 | (1.45, 16.12) |  |
|  |  | White non-Hispanic and Other | 14 | 4.79 | (-2.55, 12.12) |  |
|  | | | | | | |
| Age (years) | Fasting Glucose (mg/dL) Change, Week 24 to Week 48 | <40 | 7 | 4.14 | (-6.47, 14.76) | 0.97 |
|  |  | 40-60 | 30 | 5.43 | (0.31, 10.56) |  |
|  |  | >60 | 5 | 4.40 | (-8.16, 16.96) |  |
|  | | | | | | |
| Semaglutide Adherence | Fasting Glucose (mg/dL) Change, Week 24 to Week 48 | 100% | 37 | 5.49 | (0.94, 10.03) | 0.62 |
|  |  | <100% | 5 | 2.20 | (-10.16, 14.56) |  |
|  | | | | | | |
| Week 24 Weight Response | Fasting Glucose (mg/dL) Change, Week 24 to Week 48 | >2.27 kg lost | 32 | 6.72 | (1.93, 11.50) | 0.17 |
|  |  | >2.27 kg not lost | 10 | -0.10 | (-8.66, 8.46) |  |
|  | | | | | | |
| Week 24 Quartile | Fasting Glucose (mg/dL) Change, Week 24 to Week 48 | Q1 | 13 | 11.69 | (4.39, 19.00) | 0.11 |
|  |  | Q2 | 10 | 5.40 | (-2.93, 13.73) |  |
|  |  | Q3 | 9 | 3.00 | (-5.78, 11.78) |  |
|  |  | Q4 | 10 | -1.90 | (-10.23, 6.43) |  |
|  | | | | | | |

Supplemental Table 4. Change in HbA1c by Subgroup

| **Covariate** | **Outcome** | **Subgroup** | **n** | **Estimated Effect** | **95% CI** | **P-value** |
| --- | --- | --- | --- | --- | --- | --- |
| Sex at birth | Glycosylated Hemoglobin A1C (%) Change, Week 24 to Week 48 | Female | 16 | 0.23 | (0.08, 0.37) | 0.98 |
|  |  | Male | 27 | 0.22 | (0.11, 0.34) |  |
|  | | | | | | |
| Gender | Glycosylated Hemoglobin A1C (%) Change, Week 24 to Week 48 | Cis or Trans Female | 19 | 0.27 | (0.14, 0.41) | 0.32 |
|  |  | Cis or Trans Male | 24 | 0.18 | (0.06, 0.30) |  |
|  | | | | | | |
| Race/Ethnicity | Glycosylated Hemoglobin A1C (%) Change, Week 24 to Week 48 | Black (regardless of ethnicity) | 16 | 0.11 | (-0.03, 0.26) | 0.14 |
|  |  | White Hispanic | 14 | 0.32 | (0.17, 0.48) |  |
|  |  | White non-Hispanic and Other | 13 | 0.25 | (0.09, 0.41) |  |
|  | | | | | | |
| Age (years) | Glycosylated Hemoglobin A1C (%) Change, Week 24 to Week 48 | <40 | 8 | 0.07 | (-0.13, 0.28) | 0.18 |
|  |  | 40-60 | 31 | 0.27 | (0.17, 0.38) |  |
|  |  | >60 | 4 | 0.13 | (-0.17, 0.42) |  |
|  | | | | | | |
| Semaglutide Adherence | Glycosylated Hemoglobin A1C (%) Change, Week 24 to Week 48 | 100% | 37 | 0.21 | (0.11, 0.31) | 0.50 |
|  |  | <100% | 6 | 0.30 | (0.06, 0.54) |  |
|  | | | | | | |
| Week 24 Weight Response | Glycosylated Hemoglobin A1C (%) Change, Week 24 to Week 48 | >2.27 kg lost | 33 | 0.25 | (0.15, 0.35) | 0.26 |
|  |  | >2.27 kg not lost | 10 | 0.13 | (-0.06, 0.32) |  |
|  | | | | | | |
| Week 24 Quartile | Glycosylated Hemoglobin A1C (%) Change, Week 24 to Week 48 | Q1 | 10 | 0.30 | (0.14, 0.46) | 0.003 |
|  |  | Q2 | 10 | 0.29 | (0.13, 0.45) |  |
|  |  | Q3 | 11 | 0.36 | (0.21, 0.52) |  |
|  |  | Q4 | 12 | -0.03 | (-0.17, 0.12) |  |
|  | | | | | | |

**Supplemental Table 5**. Characteristics Associated with Weight Regain (defined as >2.27 kg) between Weeks 24-48 Among the 34 Participants with Initial Significant Weight Loss (defined as ≤-2.27 kg) from Weeks 0-24.

|  | | **Significant Regain?** | |  |
| --- | --- | --- | --- | --- |
| **Characteristic** | **No (N=14)** | | **Yes (N=20)** | **P-value** |
| Age (Years) | 48 (42, 52) | | 53 (41, 60) | 0.31 ^a^ |
| Sex at birth |  | |  |  |
| Female | 5 (36%) | | 9 (45%) | 0.73 ^b^ |
| Male | 9 (64%) | | 11 (55%) |  |
| Race/Ethnicity |  | |  |  |
| Black (regardless of ethnicity) | 3 (21%) | | 8 (40%) | 0.31 ^b^ |
| White Hispanic | 7 (50%) | | 5 (25%) |  |
| White non-Hispanic and Other | 4 (29%) | | 7 (35%) |  |
| Baseline BMI (kg/m^2^) | 34.6 (31.1, 38.8) | | 33.8 (31.3, 38.4) | 0.90 ^a^ |
| Weight Change (kg), Baseline to Week 24 | -9.8 (-12.6, -5.2) | | -11.0 (-13.1, -6.2) | 0.82 ^a^ |
| *Diet Summary Score (0-27)* |  | |  |  |
| Baseline | 11.0 (10.0, 15.0) | | 10.5 (8.0, 13.0) | 0.58 ^a^ |
| Change, Baseline to Week 24 | -1.0 (-4.0, 0.0) | | -1.0 (-3.0, 1.5) | 0.38 ^a^ |
| Change, Week 24 to Week 48 | 0.5 (-1.0, 3.0) | | 0.0 (-1.0, 3.0) | > 0.999 ^a^ |
| Change, Baseline to Week 48 | -0.5 (-2.0, 1.0) | | 0.0 (-2.0, 2.0) | 0.61 ^a^ |
| *Walk MET-minutes/week* |  | |  |  |
| Baseline | 693.0 (132.0, 1386) | | 1386 (495.0, 3465) | 0.038 ^a^ |
| Change, Baseline to Week 24 | 0.0 (-198, 396) | | 66.0 (0.0, 1188) | 0.29 ^a^ |
| Week 24 | 511.5 (132.0, 1188) | | 1056 (462, 2772) | 0.048 ^a^ |
| Change, Week 24 to Week 48 | 99.0 (-231, 396) | | -132.0 (-1205, 841.5) | 0.36 ^a^ |
| Week 48 | 660.0 (264, 990) | | 1683 (396.0, 2772) | 0.18 ^a^ |
| Change, Baseline to Week 48 | 82.5 (-99.0, 528.0) | | -99.0 (-1584, 1485) | 0.35 ^a^ |
| *Moderate MET-minutes/week* |  | |  |  |
| Baseline | 0.0 (0.0, 360.0) | | 640.0 (120.0, 3000) | 0.021 ^a^ |
| Baseline to Week 24 | 0.0 (-120, 720.0) | | 600.0 (0.0, 2400) | 0.24 ^a^ |
| Week 24 | 120.0 (0.0, 1200) | | 2640 (320.0, 3600) | 0.041 ^a^ |
| Change, Week 24 to Week 48 | 0.0 (0.0, 120.0) | | -2040 (-3000, 360.0) | 0.15 ^a^ |
| Week 48 | 600.0 (40.0, 840.0) | | 720.0 (160.0, 1440) | 0.38 ^a^ |
| Change, Baseline to Week 48 | 40.0 (-240, 840.0) | | 0.0 (-1440, 560.0) | 0.35 ^a^ |
| *Vigorous MET-minutes/week* |  | |  |  |
| Baseline | 0.0 (0.0, 720.0) | | 160.0 (0.0, 1440) | 0.52 ^a^ |
| Baseline to Week 24 | 0.0 (-280, 0.0) | | 0.0 (-240, 2160) | 0.36 ^a^ |
| Week 24 | 180.0 (0.0, 1440) | | 120.0 (0.0, 2880) | 0.71 ^a^ |
| Change, Week 24 to Week 48 | 0.0 (-960, 0.0) | | 0.0 (-2520, 160.0) | >0.999 ^a^ |
| Week 48 | 0.0 (0.0, 320.0) | | 80.0 (0.0, 2880) | 0.29 ^a^ |
| Change, Baseline to Week 48 | 0.0 (-720, 240.0) | | 0.0 (-160, 1280) | 0.39 ^a^ |
| *Total physical activity MET-minutes/week* |  | |  |  |
| Baseline | 1485 (693.0, 2412) | | 3564 (1902, 6786) | 0.0064 ^a^ |
| Change, Baseline to Week 24 | 300.0 (-705, 1242) | | 1170 (-165, 4706) | 0.19 ^a^ |
| Week 24 | 1617 (1017, 2106) | | 4230 (1884, 9198) | 0.016 ^a^ |
| Change, Week 24 to Week 48 | -121 (-840, 1386) | | -1269 (-4725, 906.0) | 0.15 ^a^ |
| Week 48 | 1380 (737.0, 3266) | | 3652 (1386, 5820) | 0.072 ^a^ |
| Change, Baseline to Week 48 | 830.0 (-1498, 1981) | | -892 (-2124, 2409) | 0.71 ^a^ |

MET, metabolic equivalent of task

Values presented as Median (IQR) or n (frequency)

^a^ Wilcoxon rank sum test

^b^ Fisher’s exact test

Supplemental Figure 1: Cross-sectional measurements at each time point for A) total cholesterol, B) LDL cholesterol, C) HDL cholesterol, D) triglycerides, E) systolic blood pressure. The red circles indicate median and red error bars indicate the interquartile range. At each study week, individual participant measurements are shown as points and are connected with grey lines to summarize within-participant changes. Violin plots illustrate the distribution and density of the cross-sectional data. Medians and interquartile ranges are shown in red with the medians joined to depict overall trend.


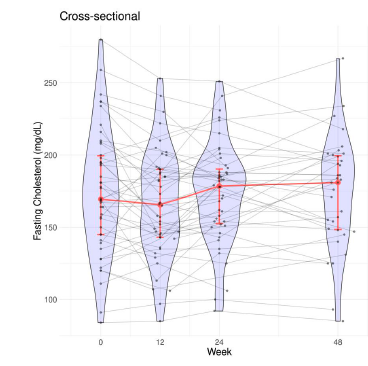


A. Total cholesterol


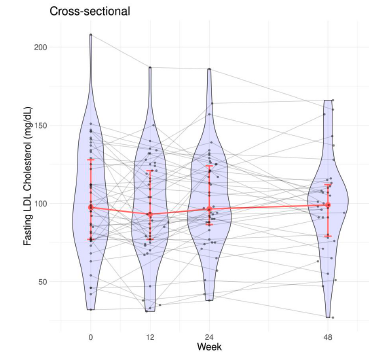


B. LDL cholesterol


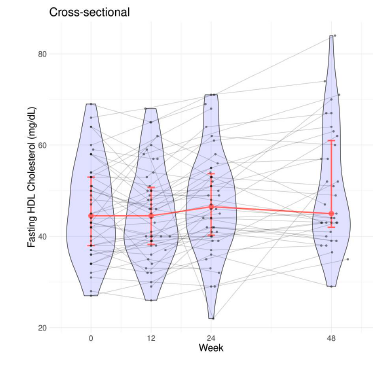


C. HDL cholesterol


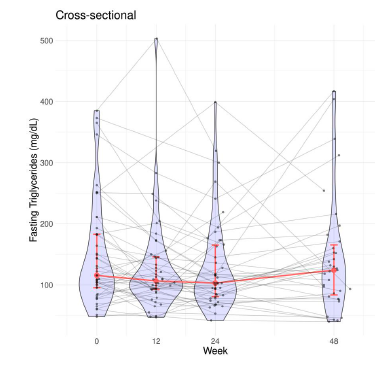
D. Triglycerides

E. Systolic blood pressure


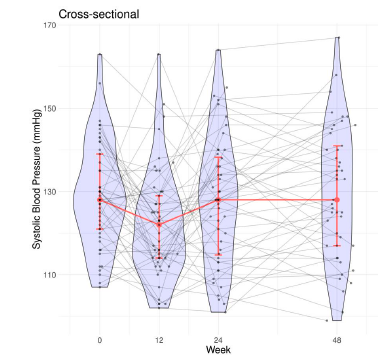

Supplement: ciaf652_Supplementary_Data [file ciaf652_supplementary_data.docx]
